# The Association Between Prosocialness, Relational-Interdependent Self-construal and Gender in Relation to Burnout Among Swedish Clergy

**DOI:** 10.1007/s13644-020-00420-3

**Published:** 2020-07-01

**Authors:** Sandra Buratti, Martin Geisler, Carl Martin Allwood

**Affiliations:** Department of Psychology, University of Gothenburg, Box 500, 405 30 Gothenburg, Sweden

**Keywords:** Individual differences, Burnout, Prosocialness, Relational-interdependent self-construal, Clergy

## Abstract

Serving as a clergyperson is a highly variable profession and in recent decades, the role has evolved and expanded even further. Consequently, the demands have increased and with it the risk for stress-related ill-health and absenteeism. The aim of the current study was to evaluate, in a larger sample of Swedish clergy (N = 871), two possible antecedents of burnout, namely prosocialness and relational-interdependent self-construal. A further aim was to explore potential gender differences in the investigated associations. The direct and indirect relationships of prosocialness and relational-interdependent self-construal to two dimensions of burnout, exhaustion, and disengagement were investigated in a structural equation-modelling framework. The results showed that clergy who reported higher prosocialness experienced more stress in their work, in terms of both quantitative and emotional demands, which in turn was associated with higher levels of exhaustion and disengagement. But prosocialness was also found to be directly associated with lower levels of disengagement, as well as indirectly associated with higher levels of role clarity. However, no direct or indirect associations were found between relational-interdependent self-construal and any dimension of burnout. Regarding gender differences, female clergy reported higher levels of prosocialness and job demands, less role clarity, and in turn more exhaustion compared to male clergy. This indicated a more stressful situation for female clergy. Our study contributes new insights into the role that personality plays in different dimensions of burnout in clergy, as well as insights into an understanding of gender differences in burnout among clergy.

## Introduction

Clergy is a highly variable profession with many potential stressors. The profession not only entails the preaching of the gospel and the delivery of rituals, but also organization, administration, and pastoral care for people in need (e.g., Milstein et al. 2005, 2010). Furthermore, providing spiritual guidance and pastoral care can be emotionally demanding and clergy are frequently approached to take on tasks typically performed by counsellors and mental health workers (e.g., Rudolfsson and Milstein 2019; Wang et al. 2003). Consequently, the clergy profession has become more demanding and stressful (Hendron et al. 2012; Proeschold-Bell et al. 2015; Proeschold-Bell and Byassee 2018), and it has been noted that emotional labour, role overload, and compassion fatigue are major clergy stressors (Adams et al. 2017; Frederick et al. 2018; Kinman et al. 2011).

Recent reports suggest that stress-related ill-health and burnout is a common concern for the clergy (e.g., Frederick et al. 2018), although some previous research suggests that the Protestant churches’ clergy report higher levels of occupational stress compared to Catholic Church clergy (Weaver et al. 2002). In Sweden, where the vast majority are Protestant, clergy has been highlighted as a profession with high levels of stress-related ill-health and absenteeism (Social Insurance Report, 2014: 4).

In line with the notion that aspects of emotionality, role overload, and compassion fatigue are important for understanding occupational stress in the clergy, this study investigated two possible antecedents for clergy burnout, namely prosocialness and relational-interdependent self-construal, among Protestant clergy serving in the Church of Sweden. In brief, prosocialness is the willingness to care for or assist others (Caprara et al. 2005), whereas relational-interdependent self-construal refers to the tendency to define oneself through one’s social roles and relationships (Cross et al. 2000). The relevance of attending to prosocialness and relation-interdependent self-construal is indicated by reports that clergy do not tend to differentiate between their personal lives and their professional role (Beebe 2007), and that this strong identification with their professional role seems to be associated with less disengagement but higher exhaustion (Innstrand et al. 2011). Thus, prosocialness and a relational-interdependent self-construal may be associated with higher levels of strain and burnout among the clergy. Furthermore, because previous research has reported gender differences in burnout (e.g., Purvanova and Muros 2010), where women often tend to report higher exhaustion and men tend to report higher disengagement, the study therefore also investigated if differences in prosocialness and relational-interdependent self-construal could form part of the explanation for commonly found gender differences in burnout.

The current study was carried out in Sweden, often referred to as one of the most secular countries in the world. In recent decades, the Protestant Church of Sweden (Lutheran) has faced many challenges and undergone major changes. For instance, in 2000 the formal bond between the Swedish state and the Church of Sweden was ended, and since then its membership rate has been declining. However, the proportion of members in the population is still considerable (5.8 million members, or roughly 56% of the population) and the Church of Sweden continues to play a significant role in Swedish society (e.g., Kasselstrand 2015; Church of Sweden 2020). Moreover, the Church of Sweden has undergone various organizational changes. The number of parishes has decreased, as smaller parishes have been merged into larger parishes and congregations, which seems to have resulted in additional strain for clergy in terms of more administrative tasks and additional organizational responsibilities. Due to under-resourced public health services, clergy in Sweden, like clergy in other parts of the world, report being increasingly approached as free health care providers (Rudolfsson and Milstein 2019). In parallel, concerns regarding the work environment and an increase in stress-related ill-health and absenteeism have been reported among the Swedish clergy (e.g., Hansson 2006; Social Insurance Report 2014). However, insights into possible antecedents to stress-related ill-health and burnout among Swedish clergy are limited and more research is needed.

In recent decades, research on burnout has grown immensely (Eurofound 2018; Schaufeli et al. 2009). Initially, burnout was considered to be a psychological response to chronic stress expressed by people who work within human services professions (Maslach and Jackson 1981; Maslach 1992). However, burnout is recognized as a possible response in all types of occupations, and the dimensionality of the concept has been the subject of extensive debates (e.g., Schaufeli et al. 2009). Furthermore, research suggests that the two core dimensions of burnout are exhaustion, referring to a depletion of mental and physical energy due to work-related strain and disengagement (c.f. depersonalization), characterized by feelings of cynicism toward one’s work and one’s clients (e.g., Qiao and Schaufeli 2011).

The existing research concerning burnout levels among clergy shows mixed results. For instance, in one study clergy were found to report higher levels of exhaustion but lower levels of disengagement (e.g., Innstrand et al. 2011). However, other studies have reported that, despite high levels of stressors such as emotional demands and role overload, clergy seem to exhibit moderate levels of burnout as compared to other professions (e.g., Adams et al. 2017). In addition, research has reported differences in the levels of burnout among clergy, with younger clergy (e.g., Francis et al. 2004) as well as female clergy reporting higher levels of burnout (e.g., Innstrand et al. 2011).

Although previous research on the antecedents of burnout has focused on work-related determinants and only given limited attention to individual differences such as personality (Alarcon et al. 2009; Swider and Zimmerman 2010), it was noted by Francis et al. (2004) that personality is important to consider in understanding clergy burnout. Regarding the role of personality, clergy who report high neuroticism and low extraversion have been found to experience higher rates of burnout (e.g., Francis and Crea 2015; Turton and Francis 2007). Francis and Crea (2015) proposed that extroverts are better prepared to meet the interpersonal expectations of the clergy profession. Moreover, in a study among clergy from several denominations, Beebe (2007) found that clergy who experienced less differentiation between the self and their profession, higher role overload, or higher role ambiguity, also reported higher indicators of risk of developing burnout. In a study of Methodist clergy, a high level of self-compassion was found to be associated with less exhaustion (Barnard and Curry 2012). Nevertheless, as the previous research on the role of personality in relation to burnout among clergy is limited, more research is needed.

Though burnout is a problem in various types of professions, workers within human services occupations seem to be especially susceptible, due to the emotional and caregiving aspects of these types of occupations (e.g., Hasenfeld 2010; Maslach et al. 2001). Indeed, it has been suggested that clergy are at risk of burnout due to the human services aspects of their profession (e.g., Frederick et al. 2018). Furthermore, it has been noted that the lower levels of disengagement observed among clergy may need to be understood in relation to their strong identification (e.g., religious identity, calling) with the job (Beebe 2007; Innstrand et al. 2011). Related to this, it has been suggested that people who have an altruistic motivation to work for the good of other people, and to serve the interests of a community (e.g., the church/congregation), are better able to deal with stressors and stay engaged because of their prosocial motivation (Bakker 2015).

Prosocialness is a trait-like disposition referring to the willingness to care for and be ready to help and assist others (Caprara and Pastorelli 1993; Caprara et al. 2005). It has been reported that engaging in work-related prosocial behaviour increases the meaningfulness a person experiences in their work (Allan et al. 2018), and prosocialness has been found to have a positive correlation with a person’s general well-being (Caprara et al. 2005). Nevertheless, being overly engaged in prosocial behaviour may increase stress and, in turn, risk having adverse effects on well-being (Windsor et al. 2008) and work-related outcomes (Bolino and Grant 2016). Illustrative of this, some studies suggest that when people feel obliged to help others they may risk taking on too much responsibility for other people, which can lead to work overload and fatigue (e.g., Grant 2008; Bolino and Turnley 2005). Furthermore, the relational-interdependent self-construal concept, which is related to prosocialness, refers to a person’s identity in relation to their close relationships. People with higher relational-interdependent self-construal experience a close connection to others, in the sense that important relationships and social roles are an intimate and important part of their definition of self. To maintain and enhance a relational-interdependent self-construal, a person may engage in prosocial acts in order to enhance their relationships and connectedness with others (Cross et al. 2000). Hence, it may be that individuals high in prosocialness—in a profession where the needs of the clients are great—will take on a greater workload and become more engaged in the emotional aspects of their work. Hence, clergy with higher prosocialness and relational-interdependent self-construal may be inclined to invest more time and effort in their work and are less likely to turn away clients in need, which in turn may blur the boundaries of their professional role. Similarly, these members of the clergy may become more involved and engaged in the needs of their family and friends, which also could have an impact on their work and their well-being (Netemeyer et al. 1996).

Thus, prosocialness and a relational-interdependent self-construal may be related to higher levels of exhaustion and disengagement by being associated with a more extensive work load, less role clarity, and more frequent experiences of personal-to-work conflict. However, it may also be that prosocialness can foster a greater sense of meaning in one’s work, thereby counteracting feelings of cynicism and disengagement (e.g., Bakker 2015; Innstrand et al. 2011). Experiencing meaning in one’s work is important for workers’ health and well-being, and is also important to the risk of burnout (e.g., Schnell et al. 2013), and recent research even suggests a causal link between prosocial behaviour and meaning in one’s work (Allan et al. 2018).

A secondary and complementary aim of the present study was to investigate if differences in prosocialness and relational-interdependent self-construal between men and women can add insights that help explain why there are gender differences in levels of burnout among clergy. In their large meta-analyses, Purvanova and Muros (2010) noted that only a few studies have investigated the relationship between gender and burnout, and found that exhaustion was slightly more associated with women whereas disengagement was somewhat more associated with men. Gender effects are of interest in relation to burnout since the conditions under which men and women live are quite different (e.g., Matud 2004), and because these conditions also differ in different occupations and societies (Purvanova and Muros 2010). For example, a recent study from Sweden showed that victims of sexual abuse more frequently sought out female clergy for pastoral care as they seemed more approachable (Rudolfsson and Tidefors 2015). This, together with the notion that clergy are being seen more frequently as potential mental health care providers (e.g., Rudolfsson and Milstein 2019) may mean an increased risk of more strain for female clergy.

In parallel, in a Norwegian study that investigated gender differences in burnout across several occupations, female clergy were found to report higher levels of exhaustion and disengagement than their male colleagues (Innstrand et al. 2011). Overall, gender differences in prosocialness may be one factor that contributes to the gender differences in burnout statistics (Eurofound 2018). Previous studies have found differences in the prosocial behaviour exhibited by men and women (Caprara et al. 2005; Diekman and Clark 2015). Women tend to exhibit more empathy and offer more emotional support to others, especially in the context of close and long-term relationships. Moreover, women are also more willing to engage in efforts to help socially disadvantaged people, whereas men are more prone to engage in providing immediate assistance that demands concrete action. In line with this, women tend to report higher relational-interdependent self-construal (Cross and Madson 1997). Furthermore, a study that investigated the role of empathy in relation to burnout among medical students found that women experienced more empathic concerns, as well as higher levels of personal distress and emotional exhaustion, compared to men (Paro et al. 2014). Thus, gender differences in sociality in respect of burnout is an area that needs further exploration.

### Hypotheses

Based on the literature and the considerations described above, we therefore hypothesized that:

#### **Hypothesis 1**

Prosocialness and relational-interdependent self-construal will have an indirect relationship with burnout as a result of its association with higher levels of job demands, lower levels of role clarity, and higher levels of personal-to-work conflict.

#### **Hypothesis 2**

That higher levels of prosocialness and relational-interdependent self-construal are each associated with lower levels of disengagement with their work among clergy.

#### **Hypothesis 3**

Female clergy will show higher levels of prosocialness and interdependent self-construal compared to male clergy, and this will be associated with higher experienced levels of quantitative demands, emotional demands and less role clarity, which in turn will be associated with higher levels of burnout.

We also investigated possible gender differences in the respective *indirect and direct associations* between prosocialness and relational-interdependent self-construal and burnout. For example, we investigated whether the association between prosocialness and role clarity was different between female and male clergy in that it may be stronger for one group; or whether the association is positive for one group but negative for the other. However, this part of the analyses was explorative only and no hypotheses were formulated.

## Method

### Participants and Procedure

Participants were clergy working in the Church of Sweden. The data analysed and reported in the current study came from the data collected in the first wave of a larger longitudinal (three-wave) study concerning individual differences in burnout among three occupational groups: clergy, teachers, and clinical psychologists. The research was approved by the Swedish National Board of Ethics (Reg. no. 608-17). In all, 2877 email addresses to clergy were collected from the Church of Sweden and these clergy were invited to participate in the study. To be eligible for participation, the clergyperson was to have worked for at least 1 year in their profession. A total of 897 (31%) of the clergy sample started the survey, and 851 (30%) completed the survey. We followed the recommendations of Newman (2014) and included all participants who had answered at least one relevant construct in the survey; thus seven persons were excluded since they failed to do so. Twelve persons were excluded because they had worked as a clergyperson for less than 1 year, and one additional person was excluded because they stated a job which indicated that the person was not a clergyperson. Since gender information was an important part of the investigations in the current study, four persons were excluded from the data analyses because they responded “other” to the gender question and because no statistically valid inferences can be drawn from such a small sample. In addition, two persons were excluded for not providing any gender information. Thus, the final sample consisted of 871 clergy (54% females) with a mean age of 49.4 years (SD = 9.89 years). The majority worked full time (92%) and had the job title of curate (57%), which is the most common job title for clergy working within the Church of Sweden. Furthermore, 43% of the clergy reported that they had a managerial position. The sample was considered highly representative for clergy working for the Church of Sweden, even though slightly more women participated in this study, since 49% of the clergy serving in the Church of Sweden are female according to information provided by the Church of Sweden’s employer organization. Also, the mean age in the current study was marginally lower than that of the working population of clergy in the Church of Sweden (52 years).

### Measures

#### Prosocialness

The Prosocialness Scale for Adults was used (Caprara et al. 2005) to measure the tendency to act prosocially. The scale has 16 items and measures prosocial orientation in terms of helpfulness, emotional empathy, etc. An example item is “I am pleased to help my friends/colleagues in their activities”. Each item was answered on a five-point scale with respect to whether the statement was “Never/almost never true” (1), “Occasionally true” (2), “Sometimes true” (3), “Often true” (4), or “Almost always/always true” (5). A higher score indicates a higher level of prosocialness. Cronbach’s alpha was .87.

#### Relational-Interdependent Self-construal

The scale by Cross et al. (2000) was used to measure relational-interdependent self-construal. The scale consists of 11 items and measures to what extent an individual defines him/herself interdependently with others in such a way that the person feels a fundamental connectedness with others. An example item is “My close relationships are an important reflection of who I am”. The response scale ranges from “Strongly disagree” (0) to “Strongly agree” (6). Cronbach’s alpha was .88.

#### Job Demands and Work Characteristics

Job demands and work characteristics were measured using three specific scales from the validated Swedish medium-length version of the Copenhagen Psychosocial Questionnaire (Berthelsen et al. 2014, 2018; Pejtersen et al. 2010). The scales assessed *Quantitative demands* (Cronbach’s alpha = .83), *Emotional demands* (Cronbach’s alpha = .79), and *Role clarity* (Cronbach’s alpha = .83). Quantitative demands and emotional demands were assessed by four items each and rated on a five-point scale ranging from “Never/Almost never” (1) to “Always” (5). An example item for quantitative demands is “Do you get behind with your work?” and for emotional demands is “Is your work emotionally demanding?”. Role clarity was measured by three items rated on a five-point scale ranging from “To a very low degree” (1) to “To a very high degree” (5). An example item is “Do you know exactly what is expected of you in your work?”. The final scores were linearly transformed according to the following formula provided with the COPSOQ-II (1 = 0, 2 = 25, 3 = 50, 4 = 75, 5 = 100).

#### Personal-to-Work Conflict

Three items were used to measure personal-to-work conflict, that is, the extent to which demands from the respondent’s personal life were perceived as obstacles to their effectiveness at work. The items 7–9 in the scale presented by Wilson and Baumann (2015) were used: “My personal activities produce stress that makes it difficult to concentrate at work”, “My personal activities drain me of energy I need to do my job.” and “I am often too tired to be effective at work because of my involvement in personal activities.” The respondents were asked to provide information with respect to the extent that each of the items “corresponded with themselves and their situation” Each item was answered on a scale ranging from “Totally disagree” (1), “Disagree” (2), “Neither disagree nor agree” (3),“Agree” (4), to “Totally agree” (5). Cronbach’s alpha was .87.

#### Burnout

*Exhaustion* was measured using the Shirom-Melamed Burnout Questionnaire (Lundgren-Nilsson et al. 2012; Melamed et al. 1992), which has 22 items and is a self-reported measure of exhaustion. It includes four subscales: Physical exhaustion (8 items, e.g., “I am physically exhausted”), Listlessness (4 items, e.g., “I feel alert”; reverse scored), Tension (4 items, e.g., “I feel relaxed” reverse scored), and Cognitive weariness (6 items, e.g., “I feel I am not thinking clearly”). Each item is rated on a seven-point scale ranging from “Almost never” (1) to “Almost always” (7). The cut-off point for clinical levels of exhaustion is a score ≥ 4.4 (Lundgren-Nilsson et al. 2012). Cronbach’s alpha was .96.

*Disengagement* was measured using the Disengagement scale of the Oldenburg Burnout Inventory (Demerouti et al. 2003; Halbesleben and Demerouti 2005; Peterson et al. 2011). The scale consists of eight items (e.g., “It happens more and more often that I talk about my work in a negative way”) measured on a four-point scale ranging from “Totally disagree” (1) to “Totally agree” (4). Cronbach’s alpha was .80.

#### Covariates

Demographic variables such as age, years working as a clergyperson, gender (0 = female, 1 = male), and managerial position (0 = no managerial position, 1 = managerial position) were included to control for potential confounding effects.

### Statistical Analyses

**Fig. 1:**
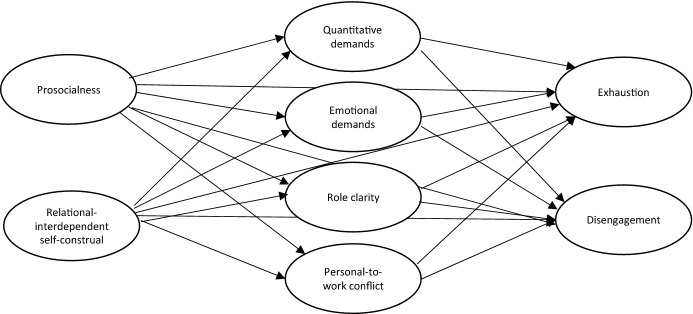
The structural equation model of direct and indirect associations between prosocialness and relational-interdependent self-construal and burnout

Descriptive statistics and Cronbach alphas were analysed using SPSS 25. Associations between prosocialness, relational-interdependent self-construal, work characteristics and job demands, as well as the two dimensions of burnout were analysed using the Mplus program 8.2. Figure 1 illustrates the estimated model. In order to investigate gender differences in the direct and indirect associations found, multi-group latent mean analyses of the causal structure were performed. Due to the sensitivity of the sample size in Chi square statistics (Chen 2007; Cheung and Rensvold 2002), additional measures of fit such as the Root Mean Square Error of Approximation (RMSEA) and the Comparative Fit Index (CFI) were also used. In order to investigate gender differences, measurement invariance needs to be established between the two groups (Byrne 2013; Chen 2007). Thus, following the standards and the procedure by Byrne (2013), the model was first established for females and males separately, and then a series of tests were performed that established sufficient measurement invariance between the two groups (Byrne et al. 1989). Full information maximum likelihood estimation was used in all models.

## Results

### Descriptive Statistics

Descriptive statistics can be found in Table 1. Clergy reported moderately higher levels of quantitative demands (*d* = 0.52), considerably higher levels of emotional demands (*d* = 1.88), and moderately lower levels of role clarity (*d* = 0.58) compared to the Danish reference norms of a working population (Pejtersen et al. 2010). The reported levels of exhaustion were also slightly higher (*d* = 0.32) than that of a large Swedish population based study (M = 2.87, the “MONICA Study”; Norlund et al. 2010). In addition, 20.3% of the clergy reported exhaustion levels above the cut-off value (4.4) for a severe level of exhaustion which is considerably higher than the proportion (13.5%) found in a Swedish working population sample (Lundgren-Nilsson et al. 2012). As can be seen in Table 1, female clergy reported higher levels of prosocialness, more quantitative demands, more emotional demands, less role clarity, and more exhaustion than male clergy. However, no significant gender differences were found for relational-interdependent self-construal, personal-to-work conflict, or disengagement. Table 2 shows the correlations between the study variables.

**Table 1:** Descriptive statistics of the study variables

	Females			Males			Total			t	*p*	Cohen’s d
	M	SD	n	M	SD	n	M	SD	n			
Prosocialness	55.58	8.36	460	54.08	7.7	399	54.88	8.09	859	2.71	.007**	0.19
Relational-interdependent self-construal	41.33	10.78	455	42.27	9.8	395	41.77	10.34	850	− 1.33	.185	− 0.09
Quantitative demands	51.23	19.22	467	48.38	17.72	404	49.91	18.58	871	2.26	.024*	0.15
Emotional demands	69.18	13.93	467	64.12	13.43	404	66.84	13.93	871	5.43	< .001***	0.37
Role clarity	60.81	19.68	465	63.92	19.12	403	62.25	19.48	868	− 2.35	.019*	− 0.16
Personal-to-work conflict	2.08	0.97	461	2.1	0.93	400	2.09	0.95	861	− 0.27	.784	− 0.01
Exhaustion	3.38	1.25	450	3.12	1.19	387	3.26	1.23	837	3.10	.002**	0.21
Disengagement	1.94	0.50	448	1.96	0.50	385	1.95	0.50	833	− 0.57	.570	− 0.04

**Table 2:** Pearson correlations for manifest variables and covariates

	PSA	RISC	QD	ED	RCL	PWC	EX	DIS	Gender	Age	YW
1. Prosocialness	–										
2. RISC	.27***	–									
3. Quantitative demands	.07*	− .03	–								
4. Emotional demands	.34***	.11***	.33***	–							
5. Role clarity	.04	.05	− .21***	− .16***	–						
6. PWC	.00	.06	.15***	.12***	− .13***	–					
7. Exhaustion	.05	− .02	.43***	.39***	− .39***	.36***	–				
8. Disengagement	− .13***	− .10**	.18***	.12***	− .42***	.22***	.53***	–			
9. Gender	− .09**	.05	− .08*	− .18***	.08*	.01	− .11**	.02	–		
10. Age	− .12***	− .07	− .09**	− .10**	.20***	− .19***	− .25***	− .19***	.14***	–	
11 YW	− .07	− .01	− .03	− .08*	.23***	− .15***	− .25***	− .14***	.21***	.72***	
12. Managerial position	− .08*	− .02	.24***	− .07	.26***	− .14***	− .16***	− .12***	.18***	.18***	.23***

*PSA* prosocialness, *RISC* relational-interdependent self-construal, *QD* quantitative demands, *ED* emotional demands, *RCL* role-clarity, *PWC* personal-to-work conflict, *EX* exhaustion, *DIS* disengagement, *YW* years working as clergy, *MP* managerial position

*p < .05, **p < .01, ***p < .001

### Multigroup Analyses of Causal Structure

Although the descriptive analyses showed that there was a difference in the mean values of several of the study variables between female and male clergy, it was also of interest to investigate whether *the associations among variables* differ between the genders. Thus, in order to investigate if there were any gender differences in the direct and indirect associations between prosocialness and relational-interdependent self-construal and burnout, multi-group analyses of the causal structure were performed. For example, the association between prosocialness and role clarity may be different for male and female clergy in such a way that it may be negative for one group while positive for the other; or the association might be stronger for one group, such that an increase in prosociality for female clergy has a larger impact on role clarity than for male clergy. If these types of differences were found, it would mean that the paths in the latent structure model (Fig. 1) differed significantly for female and male clergy. However, the results from the multi-group analyses did not find any such differences; thus the same model can be used for both female and male clergy.

### Direct and Indirect Associations Between Prosocialness and Relational-Interdependent Self-construal and Burnout

**Fig. 2:**
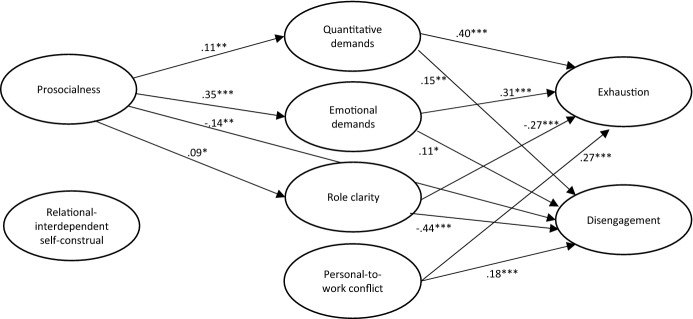
Statistically significant direct and indirect associations between prosocialness and relational-interdependent self-construal and burnout. Standardized beta values are reported. *p < .05, **p < .01, ***p < .001. χ_(381)_^2^ = 1364.33, RMSEA = .054 (90% CI = .051–.058), CFI = .929, TLI = .914

Since no gender differences were found for the direct and indirect associations between prosocialness and relational-interdependent self-construal in relation to the two dimensions of burnout, a final combined model was estimated. In this model, gender was added to the latent factors as a covariate along with age, years working as a clergyperson and managerial position. The model with the standardized effects of the latent structure is depicted in Fig. 2. The model fit was acceptable: χ_(381)_^2^ = 1364.33, RMSEA = .054 (90% CI = .051–.058), CFI = .929, TLI = .914.

The standardized direct effects of the entire model are reported in Table 3. When controlling for the other variables, male clergy experienced lower levels of quantitative and emotional demands, but more disengagement. With increasing age, clergy reported less prosocialness and relational-inter dependent self-construal and, in turn, lower levels of quantitative demands, personal-to-work conflict, and disengagement. The more years the clergyperson had worked, the more role clarity and less exhaustion they experienced. Clergy who had a managerial position experienced higher levels of quantitative demands, but also reported higher role clarity, less personal-to-work conflict, and lower levels of exhaustion.

**Table 3:** Direct associations between prosocialness and relational-interdependent self-construal variable and different aspects of burnout derived from the combined multivariate latent model

Predictor	Outcome variables															
	PSA		RISC		QD		EM		RCL		PWC		EX		DIS	
	β	SE	β	SE	β	SE	β	SE	β	SE	β	SE	β	SE	β	SE
Gender, females = 0	− 0.07	0.04	0.06	0.03	− 0.11**	0.04	− 0.15***	0.04	0.01	0.04	0.05	0.04	0.04	0.03	0.11**	0.04
Age	− 0.13**	0.05	− 0.16**	0.05	− 0.16**	0.05	− 0.04	0.05	0.11	0.06	− 0.15**	0.05	− 0.05	0.05	− 0.15**	0.05
Years working as clergy	0.06	0.05	0.07	0.06	0.03	0.05	− 0.02	0.05	0.13*	0.06	− 0.03	0.05	− 0.11*	0.05	0.06	0.04
Managerial position	− 0.06	0.04	− 0.04	0.04	0.29***	0.03	− 0.01	0.04	0.24***	0.04	− 0.13***	0.04	− 0.12**	0.04	− 0.01	0.04
Prosocialness					0.11*	0.04	0.35***	0.04	0.09*	0.04	− 0.03	0.04	− 0.07	0.04	− 0.14**	0.05
RISC					− 0.07	0.05	0.04	0.04	0.03	0.04	0.06	0.04	− 0.05	0.03	− 0.06	0.04
Quantitative demands													0.40***	0.04	0.15**	0.05
Emotional demands													0.31***	0.04	0.11*	0.05
Role clarity													− 0.27***	0.04	− 0.44***	0.04
Personal-to-work conflict													0.27***	0.04	0.18***	0.04

*PSA* prosocialness, *RISC* relational-interdependent self-construal, *QD* quantitative demands, *ED* emotional demands, *RCL* role-clarity, *PWC* personal-to-work conflict, *EX* exhaustion, *DIS* disengagement, *MP* managerial position

*p < .05, **p < .01, ***p < .001

Clergy who reported higher prosocialness also reported higher levels of quantitative demands, emotional demands, and role clarity, but also less disengagement. No relationships were found between relation-interdependent self-construal and any of the other variables. Higher levels of quantitative and emotional demands, as well as of personal-to-work conflict, were associated with higher levels of exhaustion and disengagement. However, higher role clarity was associated with lower levels of exhaustion and disengagement. No direct association was found between prosocialness and exhaustion, but there was a direct and negative association between prosocialness and disengagement. Clergy who reported higher levels of prosocialness therefore also experienced less disengagement.

In brief, the results suggest several possible indirect effects of prosocialness on burnout through work demands and role clarity. Statistical evaluations of these indirect effects suggest that prosocialness was positively correlated to exhaustion through higher levels of quantitative demands (*β *= 0.04, *SE* = 0.02, *p* = .016) and emotional demands (*β *= 0.11, *SE* = 0.02, *p* < .001). Prosocialness was also negatively correlated to exhaustion through higher levels of role clarity (*β *= − 0.03, *SE* = 0.01, *p* = .022). No significant indirect effect was found via personal-to-work conflict (*β *= − 0.01, *SE* = 0.01, *p* = .433).

A direct effect of prosocialness on disengagement was found; persons high in prosocialness reported feelings of disengagement less frequently. Prosocialness was also found to be indirectly and positively associated with disengagement through higher levels of emotional demands (*β *= 0.04, *SE* = 0.02, *p* = .015), and negatively through higher levels of role clarity (*β *= − 0.04, *SE* = 0.02, *p* = .029). No significant relationships were found between prosocialness and disengagement via quantitative demands (*β *= 0.02, *SE* = 0.01, *p* = .074) or personal-to-work conflict (*β *= − 0.01, *SE* = 0.01, *p* < .436). Moreover, no direct effects of relational-interdependent self-construal on the different dimensions of burnout were found. Neither were there any effects of relational-interdependent self-construal on different work characteristics or job demands.

## Discussion

This study evaluated two possible antecedents of burnout—prosocialness and relational-interdependent self-construal—in a sample of clergy. It also explored possible gender differences in the investigated study variables and associations between these variables. Firstly, we note that despite alarming reports concerning the high prevalence of stress-related absenteeism in this profession (the Swedish Social Insurance Agency 2014), our sample only reported slightly higher levels of exhaustion compared to a large, non-clinical, population-based sample (Norlund et al. 2010). However, one in five clergy (20%) in our sample reported clinical levels of exhaustion. This is a rather high proportion, since only 13.5% in a normal working population have reported similar levels of exhaustion (Lundgren-Nilsson et al. 2012). The high levels of emotional and quantitative demands, as well as the low levels of role clarity, may form part of the explanation for the higher proportion of clinical reports of exhaustion in our sample.

Our first hypothesis was only partly confirmed since indirect effects on burnout were only found for prosocialness through job demands and role clarity. Prosocialness was associated with higher reported levels of quantitative demands and, in particular, higher levels of emotional demands. This provides support for the notion that higher levels of prosocialness contribute to higher engagement with the needs of clients (Bakker 2015)—needs that often are highly emotional in nature (Hendron et al. 2012). Given the result that reported quantitative demands and emotional demands were associated with exhaustion, prosocialness may be a key factor in order to understand why some clergy experience more exhaustion than others. In contrast, no significant relationships were found between relational-interdependent self-construal and the different job demands in the model. When controlling for prosocialness, it appears that interdependent-relational self-construal instead taps into a dispositional view of the self that seems to be mainly unrelated to the other study constructs. It should also be noted that the correlation between prosocialness and relational-interdependent self-construal was rather small, considering the apparent theoretical closeness of the two constructs. This suggests therefore that relational-interdependent self-construal and prosocialness are not as closely related as has been previously suggested (Cross et al. 2000).

Contrary to our expectations, a positive association was found between prosocialness and role clarity. This indicates that clergy with higher prosocialness experience a clearer perception of the boundaries and goals of their work role. In turn, this may help to explain the negative relationship between prosocialness and disengagement in our results (Hypothesis 2), and is in line with reports from experimental studies showing that work-related prosocial activities increase the meaningfulness of one’s work (Allan et al. 2018). However, less disengagement might not be exclusively beneficial. To illustrate, if a clergyperson experiences high job demands, then disengagement may act as an alarm bell that reminds him or her that they need to disengage from their work in order to allow for well-needed recovery periods. In brief, prosocialness seems to both contribute to and mitigate against burnout. It may increase experienced quantitative demands and emotional demands and this may increase exhaustion. However, prosocialness also appears to be directly associated with decreased disengagement and with increased role clarity, which was also found to be related to decreased disengagement. In order to utilize the positive effects and mitigate the negative effects of prosocialness on burnout, interventions which aim to increase awareness of the different potential effects of prosocialness should be developed for clergy in danger of burnout. Such interventions might be to discuss in order to clarify what would be reasonable boundaries and the content of the professional role of the clergy. Having clear boundaries may help clergy to not over-engage with their clients and in turn may decrease their perceived workload.

In addition, and contrary to what might be anticipated, neither prosocialness nor relational-interdependent self-construal were found to be significantly related to reports of personal-to-work conflict. There could be several possible explanations for this. Firstly, as measured in the present study, prosocialness may relate more strongly to prosocial values and behaviour directed towards non-family members. Secondly, as measured in the present study, the personal-to-work conflict items can assess if *any type* of personal life matter is an obstacle to a person’s effectiveness at work. Thus, such matters do not necessarily need to have a link to prosocialness but could instead be a result of other engagements.

Although there were no differences between gender in the associations between prosocialness and relational-interdependent self-construal and burnout, respectively, we did find, in line with our expectations (Hypothesis 3), observed mean value differences in prosocialness, job demands, role clarity, and exhaustion. However, although female clergy on average reported higher levels of prosocialness and quantitative demands as well as lower levels of role clarity compared to their male colleagues, the gender differences were quite small. The largest gender difference was found for the reported levels of emotional demands. While further research is needed to understand why female clergy experience higher levels of emotional demands than their male colleagues do, it could be that female clergy are more often contacted by parishioners in need. In support of this, a study by Rudolfsson and Tidefors (2015) found that individuals who had suffered from sexual abuse expressed that they would rather seek out female clergy as providers of pastoral care, as they were seen as less threatening and easier to confide in. However, the results of our study also highlight another possible explanation for the gender differences in exhaustion that have been reported in the literature (e.g., Purvanova and Muros 2010). Our results show an association between having a managerial position and lower levels of exhaustion. Thus, it seems that the gender differences in exhaustion may, in part, be explained by the fact that female clergy hold managerial positions less frequently than their male colleagues do. But the difference may also be explained partly by the fact that female clergy have worked fewer years than their male colleagues, since the more years the clergyperson had worked, the less exhaustion they reported and the older the clergyperson, the less disengagement they reported. Nonetheless, although the gender difference in the mean levels of exhaustion was rather small, the observed difference is in line with what has been reported in previous research among clergy (Innstrand et al. 2011).

### Strengths, Limitations, and Future Directions

A strength of the present study is that it is based on a representative and large sample of Swedish clergy. It should also be noted that even though the context for this study is Sweden, we believe that its results would at least be generalizable to clergy in other Protestant or Anglican denominations within Westernized societies. However, it is not unlikely that this study’s associations between prosocialness and burnout would also hold for other groups and denominations. This is based on the notion that clergy across different denominations have many similar duties, one of which is to meet with and provide pastoral care for people in great need (e.g., Milsten et al. 2005, 2010).

Our study contributes new insights into the role of personality in different dimensions of burnout in clergy, as well as insights into an understanding of gender differences in burnout among clergy. It should be noted however that the data in the present study is cross-sectional. Thus, no causal relationships can be inferred. It would therefore be interesting to investigate further the associations between prosocialness and burnout among clergy in a longitudinal study. Such a study could address whether an association between prosocialness and lower levels of disengagement is beneficial in the long term, or if it leads clergy to press on and therefore is associated with higher levels of exhaustion over time. Another interesting topic would be to investigate the differences in the tasks performed by female and male clergy. As indicated by Rudolfsson and Tidefors (2015), it is possible that female clergy engage in more emotionally demanding tasks, which may further explain the differences in exhaustion among female and male clergy.
